# A signal processing method for alignment-free metagenomic binning: multi-resolution genomic binary patterns

**DOI:** 10.1038/s41598-018-38197-9

**Published:** 2019-02-15

**Authors:** Samaneh Kouchaki, Avraam Tapinos, David L. Robertson

**Affiliations:** 1 Evolution and Genomic Sciences, School of Biological Sciences, Faculty of Biology, Medicine and Health, The University of Manchester, Manchester, M13 9PT UK; 2 Department of Engineering Science, University of Oxford, Oxford, OX3 7DQ UK; 3 MRC-University of Glasgow Centre for Virus Research, Glasgow, G61 1QH UK

## Abstract

Algorithms in bioinformatics use textual representations of genetic information, sequences of the characters A, T, G and C represented computationally as strings or sub-strings. Signal and related image processing methods offer a rich source of alternative descriptors as they are designed to work in the presence of noisy data without the need for exact matching. Here we introduce a method, multi-resolution local binary patterns (MLBP) adapted from image processing to extract local ‘texture’ changes from nucleotide sequence data. We apply this feature space to the alignment-free binning of metagenomic data. The effectiveness of MLBP is demonstrated using both simulated and real human gut microbial communities. Sequence reads or contigs can be represented as vectors and their ‘texture’ compared efficiently using machine learning algorithms to perform dimensionality reduction to capture eigengenome information and perform clustering (here using randomized singular value decomposition and BH-tSNE). The intuition behind our method is the MLBP feature vectors permit sequence comparisons without the need for explicit pairwise matching. We demonstrate this approach outperforms existing methods based on k-mer frequencies. The signal processing method, MLBP, thus offers a viable alternative feature space to textual representations of sequence data. The source code for our Multi-resolution Genomic Binary Patterns method can be found at https://github.com/skouchaki/MrGBP.

## Introduction

Algorithms in bioinformatics use textual representations of genetic information, sequences of the characters A, T, G and C represented as strings or sub-strings. For example, in genome assembly, exact substring matching of short *k*-mers of fixed length are typically used to identify related sequences/strings^1,2^. Although this approach works well for closely related data, it will fail predictably with divergent sequences, e.g., viruses, due to a lack of homologous regions retaining sufficient sequence identity for exact matching. While there are approaches that permit relaxed *k*-mer matching^3,4^, the processing methods used in signal/image processing offer an alternative feature space because they are designed to be rotation and scale invariant, and are generally less sensitive to noise by mapping data to a less detailed representation, i.e., ‘texture’ changes. Due to the discriminative power and computational simplicity of such techniques, they have found applications to many areas^5^. Consequently, they may work better for divergent genome information such as found in microbial communities and in particular for viruses many of which remain uncharacterised.

**Figure 1:**
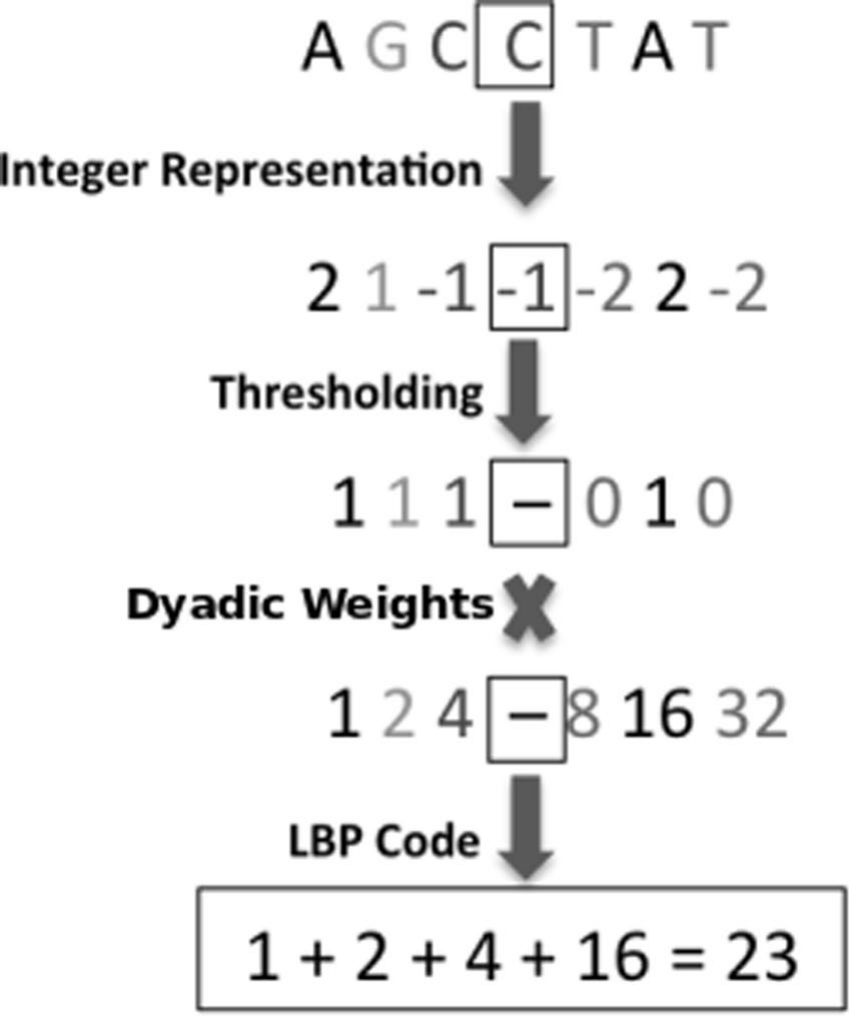
Calculating the LBP code. A threshold of the integer numerical representation of the sequence (see Table 1) is determined by comparing the centre point (in the square) and its neighbours. The LBP code is then obtained by using dyadic weights.

We have implemented a signal processing method adapted from image comparisons (local binary patterns, LBP, Fig. 1) for the extraction of local changes in numerical representations of genetic sequence data. Preliminary results have been presented as conference papers using a linear^6^ or non-linear^7^ dimensionality reduction approach. LBP is a feature descriptor capturing local texture changes first introduced for segmenting an image in two-dimensions into several meaningful partitions^8,9^. It is based on assigning a code to each local window. Its implementation for one-dimensional data has been applied to other signal processing areas, specifically, speech processing^10,11^. Here we implement the superior multi-resolution version of LBP, called multi-resolution LBP (MLBP), which considers texture changes at different scales^12^ and benchmark its use in the processing of metagenomics data. We rationalise that in the same way as images, genomic sequences have ‘texture’ patterns at various scales that can be extracted using MLBP. Crucially for alignment/homology-free comparison the arbitrary location of each pattern does not affect the extracted feature vector.

To test an application of the MBLP method and its effectiveness compared to LBP and string-based methods, we consider the problem of unsupervised grouping of genomic contigs into species-level groups (‘binning’) based on alignment-free genome composition comparisons. High-throughput/‘next-generation’ sequencing technologies have generated enormous volumes of data in metagenomic studies. In these samples, the sequence reads can be from the same or different genomes from a microbial community of viruses and bacteria, including divergent variants of the same species. Hence, reconstructing (assembling) individual genomes from this mixed data can be problematic. Moreover, sequencing errors, sequence repetition, insufficient coverage and high levels of genetic diversity can give rise to fragmented assemblies. Furthermore, comparing metagenomic data to existing reference genomes (taxonomic binning) will only identify some of the reads/contigs present. Consequently, genome composition-based techniques^13,14^ have been introduced as an alternative way to analyse the species composition of metagenomic samples^15^. These methods use species-specific genomic signatures extracted by calculating the normalised frequency of *k*-mers of a specific size, commonly *k* = 4^16,17^. The signatures are obtained by counting the occurrences of each *k*-mer combination where the *k*-mer frequency of each sequence represents a feature vector in high-dimensional space.

A number of metagenomic binning techniques have used genomic signatures as features, for example, leveraging across-sample coverage-profiles^18,19^. The method emergent self organising maps (ESOM) based binning uses contour boundaries to visualise the clusters^19^. Unfortunately, ESOM plots are computationally very demanding. Other methods that consider coverage across multiple samples include CONCOCT^18^ and MetaBAT^20^. However, they require a high number of samples to perform well, e.g., 50 or more. VizBin^17^ is another visualisation approach that considers a single sample, but it needs manual selecting of the centroids for binning.

To perform clustering/binning we have first used singular value decomposition (SVD)^21,22^ (specifically randomised SVD, RSVD^23^, for time efficiency) to reduce the dimensionality of the data, i.e., to identify the principal components of the MBLP feature vectors; termed ‘eigengenome’ information^24^. Second, these eigengenome features are passed as an input to Barnes-Hut t-distributed stochastic neighbor embedding (BH-tSNE)^25^ for visualisation of the clusters in the data.

**Figure 2:**
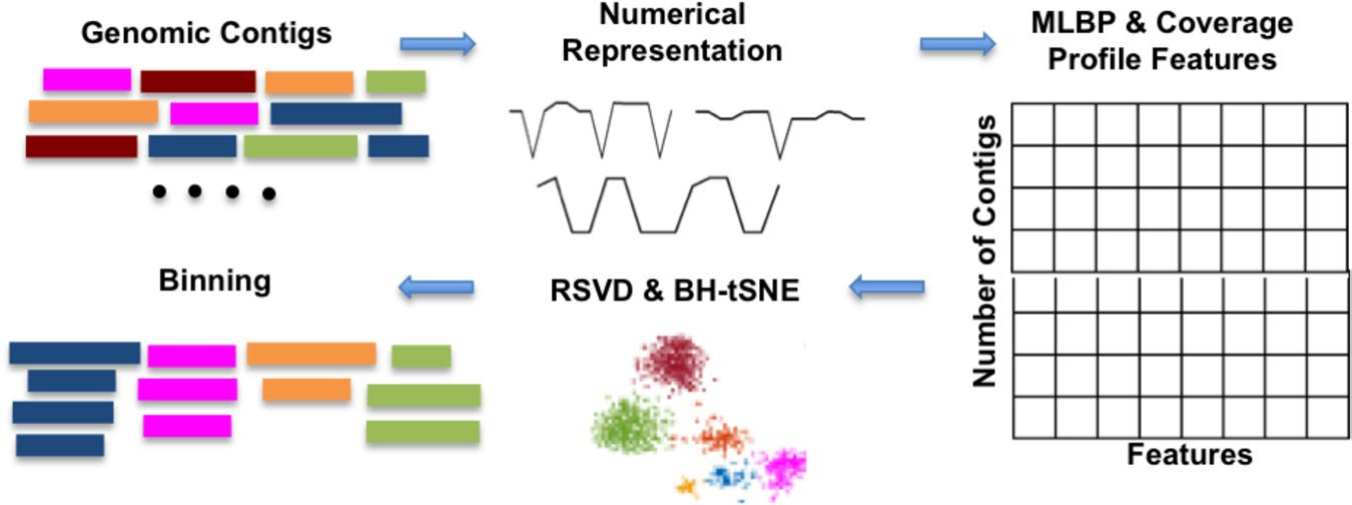
Schematic overview of our implementation of the MrGBP method to characterise the species relationships among metagenomic contigs.

An overview of our approach Multi-resolution Genomic Binary Patterns (MrGBP) is depicted in Fig. 2. We apply our method to both simulated and real metagenomic datasets, and demonstrate our results compare favourably to several existing binning methods. We also consider the effect of including coverage information across-samples in a hybrid approach to maximise the performance in longitudinal metagenomic samples and show improved performance. Collectively our results demonstrate the use of an image/signal processing method (MLBP) in bioinformatics, a new feature space for sequence analysis. The platform information for the reported run times is provided in the ‘Additional information’ Section.

## Results and Discussion

Calculating MLBP requires numerical data as an input (Fig. 1). Thus, genomic sequences need to be first mapped into one or several numerical representations^26,27^. Representation methods can be based on biochemical or biophysical properties of DNA molecules or be arbitrarily assigned numbers (Table 1). MLBP features are then extracted from these numerical representations and used to compare sequence data.

**Table 1:** The numerical representation of each letter considering Integer, EIIP, atomic and real.

Letter	Integer	EIIP	Atomic	Real
A	2	0.1260	70	−1.5
T	−2	0.1335	78	1.5
C	−1	0.1340	58	−0.5
G	1	0.0806	66	0.5

**Figure 3:**
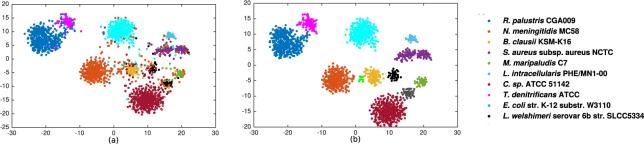
Visualisation of the simulated metagenomic community using Integer nucleotide mapping, MLBP to extract features, RSVD for feature reduction, BH-tSNE two-dimensional representation and cluster identification using DBSCAN comparing (**a**) manually annotated clusters (see species names in key) to (**b**) the DBSCAN defined clusters.

The performance of our method is tested for a low complexity simulated dataset using different numerical mappings (EIIP, atomic, real and integer nucleotide representations, Table 1) for MLBP lengths *p* ≤ 6 (Supplementary Figure 1). For example, for the integer representation our automated binning approach very closely matches the manually annotated clusters (compare panels a and b in Fig. 3). Specifically, the contigs from different species form visually separate clusters with very limited overlap with the clusters of other species.

The different numerical representations provided slightly different data clusters but overall the results demonstrated similar performance (Table 2). The Integer representation was selected for subsequent analysis as it has relatively high performance and demonstrated more discrimination compared to the other representation methods. The average run time was 75.35 seconds (2184 contigs with total length 33138556 nucleotides). The run time includes loading the data, numerically representing the data, MLBP feature extraction and dimensionality reduction using BH-tSNE (Fig. 2).

**Table 2:** Precision, recall, F1 score (%) and the number of clusters for various nucleotide mappings for a simulated low complexity metagenomic dataset.

Nucleotide mapping	Atomic	EIIP	Real	Integer
Precision	97.23	89.08	97.41	98.38
Recall	94.82	96.80	93.96	96.35
F1 score	96.01	92.78	95.65	97.35
Number of clusters	12	10	13	12

To check the effect of changing the window length, we considered various lengths of MLBP windows (Table 3 and Supplementary Figure 2). As the MLBP vectors are based on a histogram, the number of features is determined by the window length, which may affect final performance. Here, run time only includes the time to numerically represent the data and MLBP feature selection.

**Table 3:** Precision, recall, F1 score (%), the number of clusters and the run times for MLBP of various window lengths or feature dimensions (*p* ≤ *P*) and integer representation.

Window length	2	4	6	8
Feature dimension	4	20	84	212
Precision	86.08	97.78	98.38	98.21
Recall	85.80	94.04	96.35	94.63
F1 score	85.94	95.87	97.35	96.39
Number of clusters	19	16	12	11
Run time	22.43	27.89	36.22	46.14

With smaller window lengths, the resulting feature vectors cannot describe the underlying structure of the metagenomic dataset, while larger feature vectors increases the time complexity (Table 3). Hence, window size should be sufficiently large to maintain the distinctness of the signal (information regarding texture changes across various contigs).

**Figure 4:**
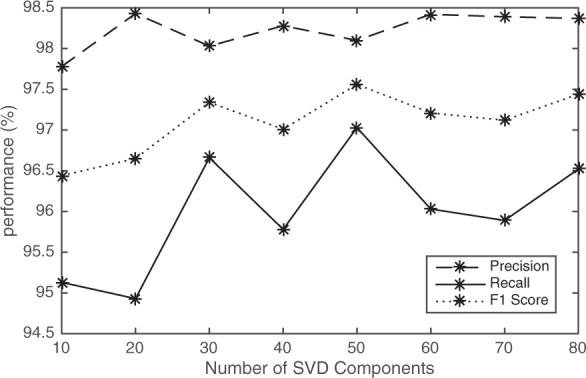
Precision, recall and F1 score (%) by keeping different numbers of RSVD components. Integer representation and *p* ≤ 6 have been considered to analyse the simulated metagenomic dataset.

The computational complexity of our method increases as the dimensions of the feature space increase. Therefore, we considered how keeping different numbers of eigen factors can affect the performance and run time of our method (Fig. 4). We use the numerical integer representation for the nucleotide mapping and *p* ≤ 6 for feature selection. The results show that after keeping a number of eigen factors, i.e., 30, the final performance does not change significantly. However, as the number of eigen factors increases the run time of RSVD and BH-tSNE increases (Table 4). These results demonstrate that the MLBP method can analyse a metagenomic dataset in a reasonable time frame. Moreover, it is performing well considering only one sample was analysed.

**Table 4:** Run times of RVSD and BH-tSNE for various number of RSVD components.

Number of RSVD Components	10	20	30	40	50	60	70	80
RSVD run time	0.06	0.12	0.20	0.30	0.44	0.55	0.69	0.85
BH-tSNE run time	27.76	28.83	30.04	31.20	33.05	34.65	35.63	35.80

### Comparison with Existing Methods for Simulated 10 and 100 Metagenomic Data

We considered two simulated datasets with 10 and 100 genomes to compare our results to both low and complex metagenomic communities. Our results compared favourably with CONCOCT^18^, MetaBAT^20^ and MaxBin2^28,29^ (Table 5). CONCOCT bins the data by employing sequence composition and across-sample coverage. The method has been compared with a range of methods including MetaWatt^30^, SCIMM^31^ and CompostBin^32^ to show its advantage over composition based techniques. However, for available high complexity metagenomic data CONCOCT does not work as well as many samples are usually required for it to perform well. MetaBAT bins the metagenomic data using probabilistic distances of genome abundance with sequence composition. It is an efficient method for analysing complex metagenomic data. MaxBin was originally introduced for single sample data in which it bins the data based on tetra-nucleotide frequencies and it has been extended to MaxBin2 to support multiple samples. MetaBAT and MaxBin2 produce many unclassified contigs. Consequently, they have higher precision but lower recalls.

**Table 5:** Precision, recall, F1 score (%) and the number of clusters for our proposed method, CONCOCT, MetaBAT and MaxBin.

Methods	Precision	Recall	F1 score	Number of clusters
10 Genomes				
MLBP	98.38	96.35	97.36	12
CONCOCT	98.56	97.35	97.95	19
MetaBAT	90.82	94.98	92.85	9
MaxBin	93.43	96.65	95.01	10
4-mer	96.14	70.80	81.54	13
LBP	90.41	96.33	93.27	11
100 Genomes				
MLBP	91.52	83.97	87.58	116
CONCOCT	60.73	96.37	74.51	79
MetaBAT	92.34	89.62	90.96	104
MaxBin	89.83	83.96	86.80	85
4-mer	95.32	69.56	80.43	98
LBP	65.60	90.67	76.13	101

For the 100 simulated genomes data our method performs better than CONCOCT and MetaBin methods, and quite close to MetaBAT. Lower performance is mainly because DBSCAN does not work very well for a very dense feature space (high complexity data representation). It may result in some unclustered contigs and therefore, lower performance. It still shows that the proposed pipeline can work for low and high complexity datasets. Note, alternative clustering methods could be explored.

For completeness we also ran our ‘MLBP pipeline’ (Fig. 2) replacing MLBP with (i) LBP and (ii) a k-mer text/string-based representation to compare our feature space with a commonly used 4-mer frequencies. The results indicate that the MLBP method has a more discriminative feature vector and better performance than either LBP or the string representation (Table 5).

### Real Data: Infant Human Gut

A relatively low-complexity infant human gut dataset^19^ was analysed to test the performance of our method with real data. A main reason for considering this dataset is to show the effectiveness of the MBLP method to bin low abundant viral community data to benchmark our texture analysis approach. The integer numerical representation was used for the nucleotide mapping, *p* ≤ 8 for feature selection and the first 60 eigen components in the dimensionality reduction stage (RSVD).

**Figure 5:**
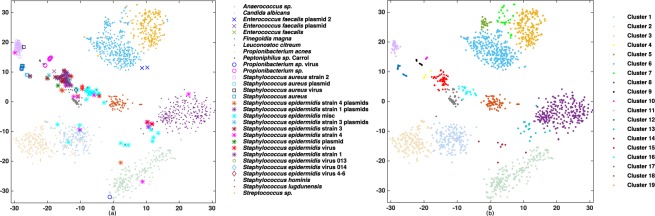
Visualisation of the infant gut metagenomic community using integer nucleotide mapping, MLBP to extract features, RSVD feature reduction, BH-tSNE two-dimensional representation and cluster identification using DBSCAN comparing (**a**) manually annotated clusters (see bacteria species, virus or plasmid names in key) to (**b**) the DBSCAN defined clusters 1 to 19.

MLBP binned the data into 19 clusters with precision and recall of 88.34 and 97.22 at the species level. BH-tSNE representation of the data demonstrates the genomic contigs of the same or very similar contigs are binned together (Fig. 5). While some of the plasmids and viruses (bacteriophages) clustered with their associated host clusters, most species formed their own cluster. The bacterial species tend to form separate clusters, for example, *Anaerococcus sp*. and *C. albicans* form clusters 1 and 3 (Fig. 5). However, separating plasmid or virus from its host is less straight-forward due to their closer genome compositions. Nonetheless, our method manages to bin *S. aureus* strains, their plasmid and virus into two groups; (1) *S. aureus* strain and plasmid and (2) *S. aureus* strain 2 and virus. *Propionibacterium sp*. appears as a separate bin. *E. faecalis* and one of its plasmids forms one cluster. *S. epidermidis* has three strains, three viruses, one integrated virus (prophage) and several plasmids and the algorithm managed to bin them into five clusters where *S. epidermidis* strains 1 and 3 clustered together (including virus 13 and 14), with strain 4 forming a separate cluster (including virus 46).

Our results compare favorably with CONCOCT^18^, MetaBAT^20^ and MaxBin2^28,29^, showing better performance on this dataset with small sample size (11 samples) in comparison with the other algorithms tested (Table 6).

**Table 6:** Precision, recall, F1 score (%) and the number of clusters for our proposed method, CONCOCT, MetaBAT and MaxBin2.

Methods	Precision	Recall	F1 score	Number of clusters
MLBP	88.34	97.22	92.57	19
CONCOCT	79.5	90.62	84.69	32
MetaBAT	84.23	92.35	88.10	10
MaxBin2	82.84	93.50	87.84	10

To further investigate the relationship between clusters, the abundance patterns of each cluster were calculated based on the number of reads mapped to contigs at the different sampling time points (Supplementary Figure 3). Pairwise correlation coefficients were then calculated to check for any pattern among the clusters. The results suggests that there is a strong correlation between clusters of related species (Supplementary Figure 3). For example, the clusters of *Propionibacterium* and *Peptoniphilus* species have similar abundance patterns (Clusters 9–10). Similar results were also found in^19^ where both species have proliferation in later stages and hence are well-adapted to the gut. Moreover, two clusters have been formed for *F. magna* with very similar coverage patterns (clusters 5-6). Consequently, this similarity could be analysed further to join some of the clusters. A similar pattern can be observed in the clusters of *S. aureus*, confirming the relationship between each bacteria and virus (clusters 11-12). The five clusters of *S. epidermidis* also share similar coverage patterns (clusters 13–17). A further step could be to cluster all the contigs of these five clusters separately to have a better separation of the related strains and viruses.

Finally, we checked the run time of our method. It takes about three minutes (108.67 s) to analyse this dataset (the number of contigs is 2293 and total length of them is 27594702). Although our code is relatively fast, it could be further optimised in terms of both time and memory.

## Conclusion

We have demonstrated that the image and signal processing technique, MBLP, can be adapted to numerical nucleotide sequence data comparisons and performs significantly better than LBP alone. Applied to metagenomic binning and visualisation, MLBP captures the genomic signature changes effectively, i.e., genome texture patterns, permitting alignment-free comparison and clustering of related contigs. Our results on simulated genomic fragments and contigs from infant human gut samples demonstrates that a signal processing method can capture the underlying taxonomic structure of the microbiome data and performs favourably in comparison to existing metagenomic methods. Collectively our results demonstrate the ‘signal’ in genome data can be just, if not more effectively, captured by appropriate image/signal processing algorithms as opposed to text/string-based methods. This demonstrates the potential for exploitation of an alternative feature space for alignment-free comparison of genomic sequence data either alone or combined with text/string-based representations, i.e., ‘multi-view’ representation of the data. Using other LBP/MLBP variants or features descriptors from image or signal processing will be investigated in future work.

## Methods

Our methodological pipeline (Fig. 2) is comprised of several steps: (1) numerically represent the genomic contigs using a nucleotide mapping (Table 1). (2) MLBP is used to extract features from these numerical representations. If available, cross-sample coverage information (mean and standard deviation) is extracted separately using Bowtie 2^33^ and can be considered as extra information to be added in the MLBP feature space. (3) Eigengenome information is extracted using RSVD to reduce the dimensions of the feature matrix. (4) BH-tSNE is used to map RSVD features to a two-dimensional space for visualisation and data binning. (5) For quantitatively evaluating the visualisation performance, we cluster the BH-tSNE projected data using DBSCAN a density-based spatial clustering algorithm^34^ and calculate the precision, recall and F1 score between the DBSCAN assigned labels and the original labels.

We note each step of the pipeline was based on having an appropriate analysis for the metagenomic data based on a novel feature space. Our primary purpose is to present this as a working view of the feature space, and not a novel metagenomics pipeline as such. Further optimisation in terms of implementation is of course possible and some options are already provided in the online software, e.g., changing the numerical representation.

### The Nucleotide Mapping

The genomic reads can be represented numerically in two ways: (i) Assigning an arbitrary value to each letter A, C, G or T of the nucleotide sequence, i.e., Voss^35^, two or four bit binary representations^36,37^ or (ii) defining numerical representations that correspond to certain biochemical or biophysical properties of the DNA molecules: electron ion interaction potential (EIIP)^38^, paired nucleotide representations^39^ or atomic representations^40^.

**Figure 6:**
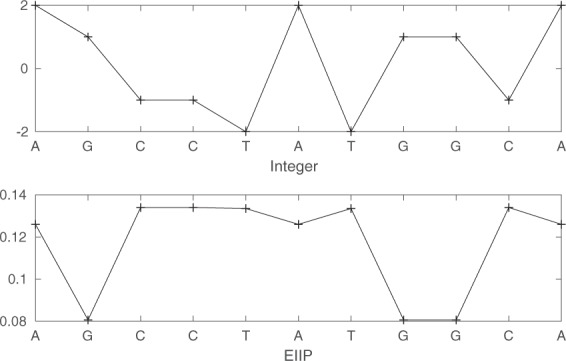
Two representations: integer and EIIP. Each nucleotide A, C, G or T in the sequence is assigned to a value depending on the numerical representation.

As each numerical representation method assigns different values to each nucleotide this can lead to different results and performance when LBP/MLBP is applied. We thus compare several existing numerical representations from the literature (EIIP, atomic, real and integer nucleotide representations). Table 1 shows the value assigned to each nucleotide in each of the representations. Figure 6 shows an example of mapping a nucleotide sequence to two numerical vectors.

### Multi-resolution Local Binary Patterns

LBP has found popularity not only in the field of image processing but also in signal processing^41^. The LBP distribution of genomic contigs was considered as the species specific genomic signatures in^6^. LBP examines the neighbouring points of each data point and assigns a binary code to it. By considering *x*(*t*) as the numerical representation of the *t* th position of a genomic segment, LBP is defined as

1 $$\begin{array}{rcl}{\rm{LBP}}(x(t)) & = & \sum _{i=0}^{p\mathrm{/2}-1}\{{\rm{Sign}}(x(t+i-p/2)-x(t)){2}^{i}\,+\\  &  & {\rm{Sign}}(x(t+i+1)-x(t)){2}^{i+p\mathrm{/2}}\},\end{array}$$

where *p* is the number of neighbouring points and Sign indicates the sign function

2 $${\rm{Sign}}(x)=\{\begin{array}{ll}0 & x < 0\\ 1 & x\ge 0\end{array}.$$

The difference between each neighbouring point and the centre point *t* is passed to a Sign function. Consequently, each window of length *p* + 1 is represented by a *p*-bit binary number. Each binary number is converted to a LBP code using a dyadic weight. An example of the LBP operator can be seen in Fig. 1 where *p* = 6. The value of the centred point (in the square in Fig. 1) is compared with the six neighbouring points to produce the binary number and LBP code. The distribution of the LBP codes are defined using the obtained codes for each window:

3 $${{\bf{h}}}_{k}=\sum _{p/2\le i\le N-p/2}\delta ({{\rm{LBP}}}_{p}(x(i),k)),$$

where *δ* shows the Kronecker delta function, *k* = 1, 2, ..., 2^*p*^ is all possible values of LBP codes and *N* is the genomic fragment length. Considering the distribution of LBP codes makes the feature space independent of each pattern location and only dependent to frequency of each MLBP code.

MLBP is an LBP extension that combines the results of LBP distribution from various values of *p* ≤ *P*. Consequently, the pattern changes of different resolution levels are considered to improve the description of the data inputs. Here, we apply MLBP to one-dimensional linear sequences to consider pattern changes of various lengths. LBP/MLBP is selected in this work due to its performance in other applications and also as it is very fast to calculate.

### Across-Samples Coverage Information

To obtain the coverage profile for contigs across the longitudinal samples, the Illumina reads were mapped to contigs with Bowtie 2^33^ for each time point. SAMtools^42,43^ was then used to produce a per base depth file. As a result, our coverage feature vector for each genomic contig is the average and standard deviation of the per base depth for each contig. Coverage information provides extra information that optionally can be added to the MLBP feature space.

### Randomised Singular Value Decomposition

A metagenomic community can be considered as a linear combination of genomic variables. The histogram of MLBP codes for each genomic fragment captures the local changes in the pattern (the “texture”) of each distinct contigs. By representing a vector of MLBP codes for each contig, low-rank matrix approximations can be used for efficient analysis of the metagenomic data. Our assumption in using SVD is that the MLBP codes of the contigs from each species have a distinct energy contribution. Therefore, the data can be represented as a linear combination of mutually independent components. RSVD a faster version of SVD was used here^23^.

SVD decomposition of a matrix **X** is defined as

4 $${\bf{X}}={\bf{U}}{\rm{\Sigma }}{{\bf{V}}}^{T},$$

where **U** and **V** are the left and right singular vectors, ∑ is singular values and (·)^*T*^ denotes the transpose operator.

In metagenomic data analysis due to data complexity, SVD can be time consuming. Therefore, RSVD is used as an accurate and robust solution to estimate the dominant eigen components quickly^23^.

RSVD calculates the first *i*th eigen components of the data by using QR decomposition and mapping **X** to a smaller matrix as

5 $$\begin{array}{rcl}{\rm{\Omega }} & = & \,{\rm{randn}}(N,i),\\ {\bf{Y}} & = & {\bf{X}}{\rm{\Omega }},{\bf{Y}}={\bf{Q}}{\bf{R}}\\ {\bf{B}} & = & {{\bf{Q}}}^{T}{\bf{X}},\end{array}$$

where randn generates a random matrix of the size of its inputs and *N* is the number of contigs. After decomposing **B** using SVD, the final factors are obtained using **Q** and the eigen factors of **B**.

### Barnes-Hut t-Distributed Stochastic Neighbor Embedding

BH-tSNE is used in many research areas as a nonlinear technique for high dimensional data visualisation^25^. It works based on keeping the locality of the data in the lower dimension and was used in this paper for two-dimensional data visualisation and clustering. BH-tSNE is based on the divergence minimisation of input objects distributions and the corresponding low-dimensional data points. As a result, it can preserve the original local data structure in the final lower dimensional visualisation. Normalised Gaussian kernel has been considered as an ordinary similarity measure but it scales quadratically to the number of data points. The main objective function also has been approximated by defining the similarity function based on a number of neighbouring points^25^. In addition, a vantage-point tree is employed for decreasing search complexity. BH-tSNE is thus an efficient (*O*(*N* log *N*)) dimensionality reduction approach and is used in this paper for two-dimensional data visualisation and clustering.

### DBSCAN

DBSCAN is a popular density-based clustering algorithm with the aim of discovering clusters from the approximate density distribution of corresponding data points. DBSCAN does not need the number of clusters to be specified but has two parameters that need to be determined: epsilon that indicates the closeness of the points of each cluster to each other and minPts, the minimum neighbours a point should have to be considered into a cluster. The initialisation point is a random point which has not been visited previously. The neighbourhood of this point is then retrieved and if it consists of an acceptable number of elements, a cluster is formed, otherwise the element is considered as noise. Hence, DBSCAN may result in some unclustered samples.

Usually DBSCAN parameters are not known prior to analysis and there are several ways to select their values. One way is to calculate the distance of each point to its closest nearest neighbour and use the histogram of distances to select epsilon. After selecting epsilon a histogram can be obtained of the average number of neighbours for each point using the epsilon. Some of the samples do not have enough neighbouring points and can be considered as noise. Implementation of the parameter selection is included in spark_dbscal (https://github.com/alitouka/spark_dbscan).

DBSCAN can find arbitrary shaped clusters, and is robust to outliers. However, it may not identify clusters of various densities or may fail if the data is very sparse. It is also sensitive to the selection of its parameters and the distance measure (usually Euclidean distance). The distance measure can affect any other clustering technique as well.

### Datasets

To validate the effectiveness of our methodology we consider both simulated and real datasets. Simulated metagenomic data of Illumina sequences for 10 and 100 genomes (Supplementary Tables 1 and 3) was downloaded from http://www.bork.embl.de/mende/simulated_data/. The data were assembled by Ray Meta^44^ into contigs (*k* = 31). Using these datasets, various aspects of our method, including MLBP window length and RSVD number of eigen components, have been analysed.

For the real data analysis, a time-series metagenomics human gut dataset comprised of 11 samples (18 runs) taken over nine days from a newborn infant^19^ was used. The authors have assembled the data into 2329 contigs. This assembly and binning information is provided at http://ggkbase.berkeley.edu/carrol/. Corresponding Illumina reads can be downloaded from the NCBI, SRA052203, which consists of 18 Illumina sequencing runs (SRR492065-66 and SRR492182-97). For the real data, we mapped the reads to the contigs using Bowtie2 and coverage profiles have been obtained using SAMtools.

### Performance Evaluation

In order to check the performance of our MrGBP method, DBSCAN^34^ has been used to cluster the final results. The precision, recall and F1 score are calculated between the DBSCAN assigned labels and the original labels to determine the performance as a measure of a clusters “purity”. Assuming there are *m* genomes in the dataset and it is binned to *k* clusters, the precision, recall and F1 score can be calculated as

6 $$\begin{array}{rcl}{\rm{Precision}} & = & \frac{\sum _{i=1}^{k}\mathop{{\rm{\max }}\,}\limits_{j}{s}_{ij}}{\sum _{i=1}^{k}\sum _{j=1}^{m}{s}_{ij}}\\ {\rm{Recall}} & = & \frac{\sum _{j=1}^{m}\mathop{{\rm{\max }}\,}\limits_{i}{s}_{ij}}{\sum _{i=1}^{k}\sum _{j=1}^{m}{s}_{ij}+\sum \,{\rm{unbinned}}\,{\rm{sequences}}}\\ {\rm{F}}1 & = & \,2\times \frac{{\rm{Precision}}\times {\rm{Recall}}}{{\rm{Precision}}+{\rm{Recall}}}\end{array}$$

where *s*_*ij*_ is the length of contigs in cluster *i* corresponds to genome *j*.

## Supplementary information

Supplementary
