# Supplementary material for: A signal processing method for alignment-free metagenomic binning: multi-resolution genomic binary patterns
Source: Sci Rep. 2019 Feb 15;9:2159. doi: 10.1038/s41598-018-38197-9 (PMC6377666; doi:10.1038/s41598-018-38197-9)
Supplement: Supplementary file 1 — Supplementary [file 41598_2018_38197_MOESM1_ESM.pdf]

A signal processing method for alignment-free metagenomic binning: multi-resolution genomic binary patterns

Samaneh Kouchaki[1,2], Avraam Tapinos[1], David L Robertson[1,3]
[1]Evolution and Genomic Sciences; School of Biological Sciences; Faculty of Biology, Medicine and Health; The University of Manchester; Manchester; M13 9PT; UK.
[2]Department of Engineering Science; University of Oxford; OX3 7DQ; UK.
[3]MRC-University of Glasgow Centre for Virus Research; Glasgow; G61 1QH; UK.

10 Genomes Metagenomic Data

Supplementary Table 1 The simulated 10 genomes data used in Results and Discussion.

Bacterial species
Rhodopseudomonas palustris CGA009
Neisseria meningitidis MC58
Bacillus clausii KSM-K16
Staphylococcus aureus subsp. aureus NCTC 8325
Methanococcus maripaludis C7
Lawsonia intracellularis PHE/MN1-00
Cyanothece sp. ATCC 51142
Thiobacillus denitrificans ATCC 25259
Escherichia coli str. K-12 substr. W3110
Listeria welshimeri serovar 6b str. SLCC5334

Supplementary Table 2 Parameter settings

minPts (DBSCAN)	epsilon (DBSCAN)	perplexity (BH-tSNE)	theta (BH-tSNE)	dims (BH-tSNE)
8	0.02	40	0.5	2

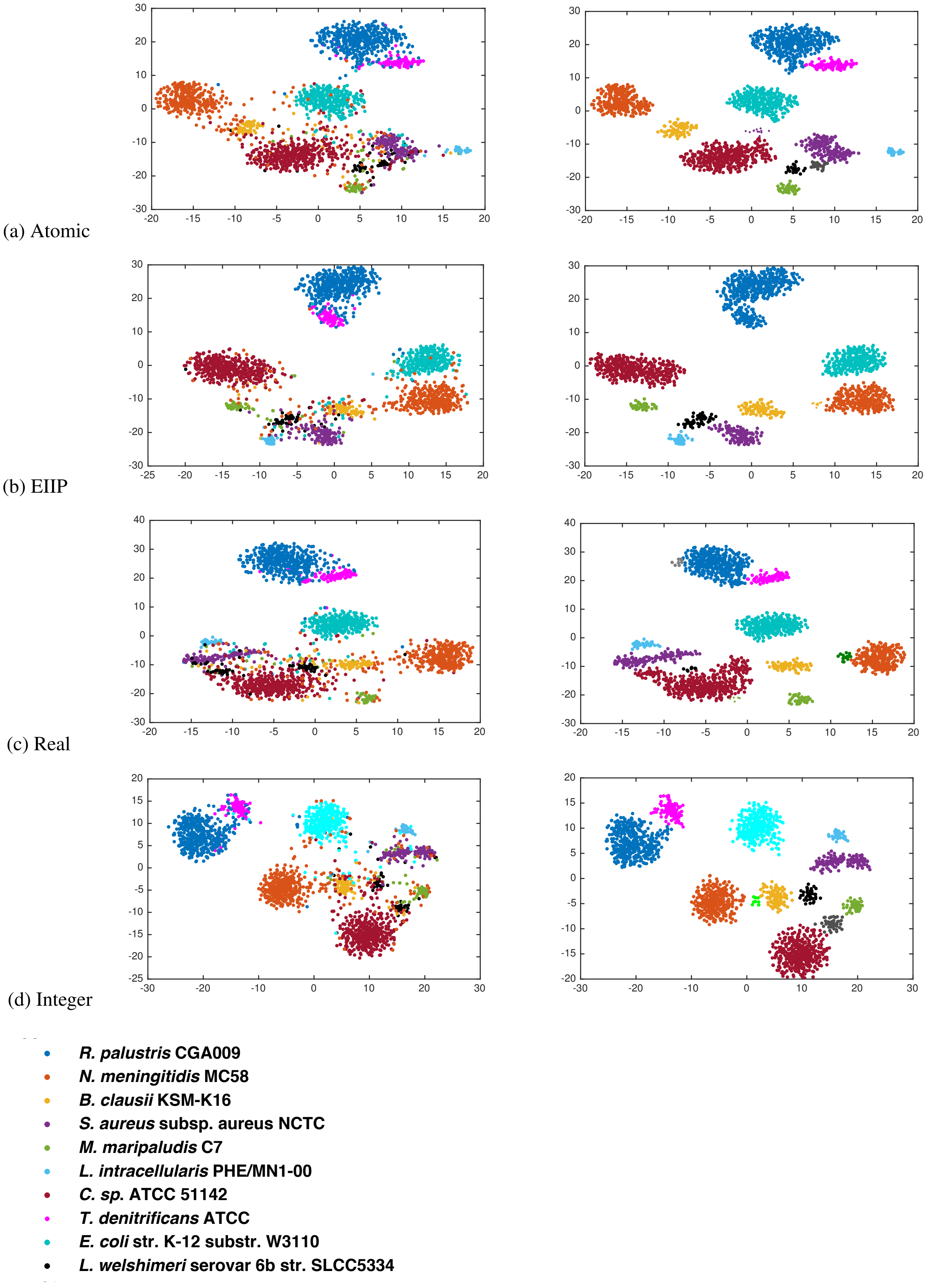

Supplementary Fig. 1 Visualisation of the simulated metagenomic community by considering various nucleotide mappings. (a) Atomic, (b) EIIP, (c) Real, and (d) Integer representation methods. Each colour represents a different species (see key) on the left side and a cluster defined by our approach on the right hand side figures.

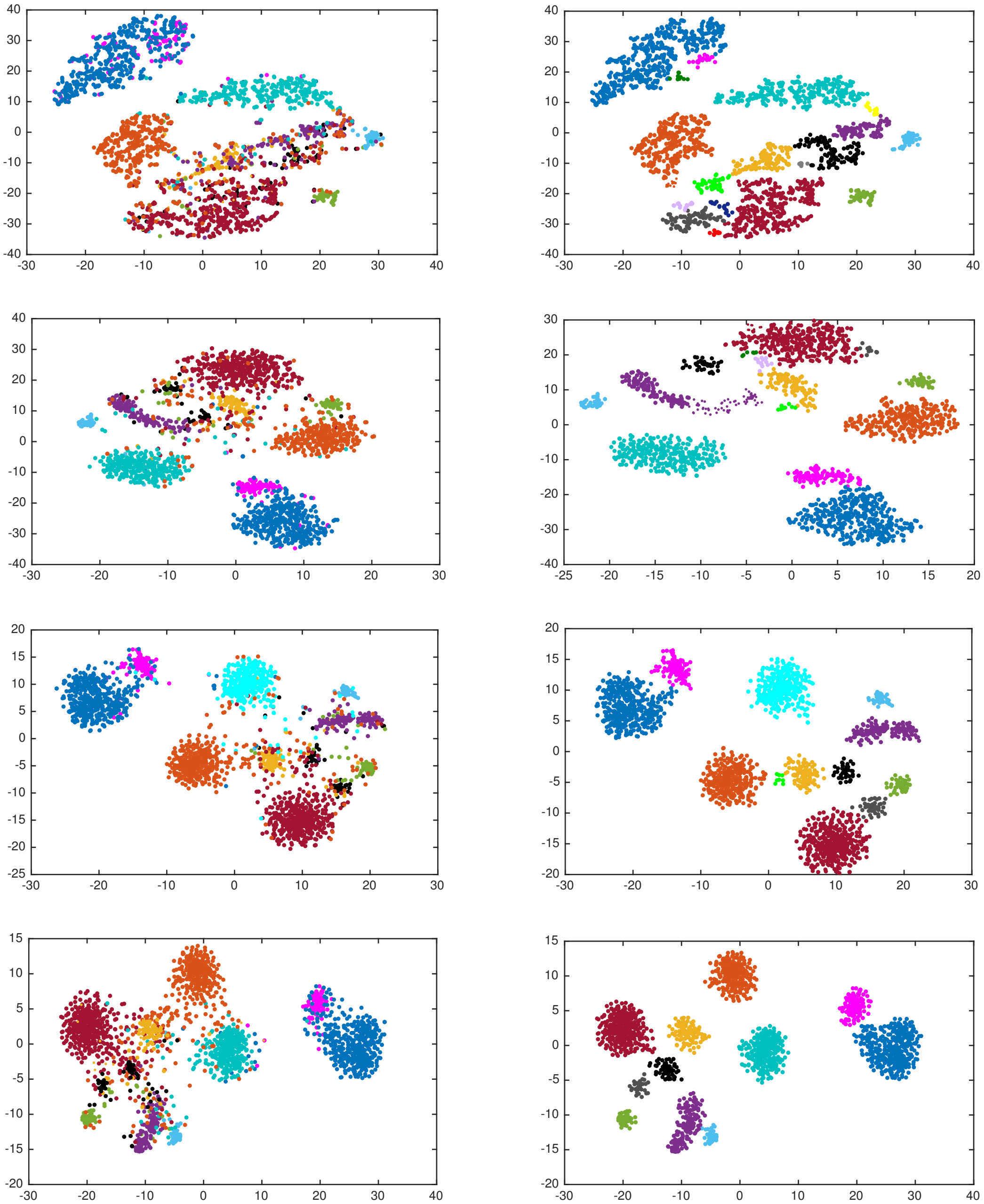

(a) $p = 2$

(b) $p \leq 4$

(c) $p \leq 6$

(d) $p \leq 8$

Supplementary Fig. 2 Visualisation of the simulated metagenomic community by considering various MLBP window lengths (a) $p = 2$, (b) $p \leq 4$, (c) $p \leq 6$, and (e) $p \leq 8$. Each colour represents a different species (see Figure 1 for key) on the left side and a cluster defined by our approach on the right hand side figures.

100 Genomes Metagenomic Data

Supplementary Table 3 The 100 genomes simulated data used in Results and Discussion.

Bacterial species
Sulfurimonas autotrophica DSM 16294
Synechococcus elongatus PCC 6301
Borrelia afzelii PKo
Paenibacillus JDR 2
Amycolicicoccus subflavus DQS3 9A1
Mobiluncus curtisii ATCC 43063
Clostridium SY8519
Teredinibacter turnerae T7901
Oligotropha carboxidovorans OM5
Candidatus Vesicomyosocius okutanii HA
Bacillus amyloliquefaciens FZB42
Aquifex aeolicus VF5
Helicobacter hepaticus ATCC 51449
Prevotella ruminicola 23
Nitrosopumilus maritimus SCM1
Methanococcus maripaludis X1
Idiomarina loihiensis L2TR
Olsenella uli DSM 7084
Dehalococcoides BAV1
Acinetobacter oleivorans DR1
Rhodoferax ferrireducens T118
Prochlorococcus marinus MIT 9211
Shewanella ANA 3
Hyphomonas neptunium ATCC 15444
Rothia dentocariosa ATCC 17931
Xylella fastidiosa M12
Clostridium botulinum Ba4 657
Francisella tularensis mediasiatica FSC147
Odoribacter splanchnicus DSM 20712
Halanaerobium hydrogeniformans
Streptococcus gallolyticus UCN34
Riemerella anatipestifer ATCC 11845
Prochlorococcus marinus MIT 9215
Clostridium difficile R20291
Desulfatibacillum alkenivorans AK 01
Thermoanaerobacter italicus Ab9
Archaeoglobus profundus DSM 5631
Sinorhizobium medicae WSM419
Staphylothermus hellenicus DSM 12710
Pyrococcus horikoshii OT3
Psychrobacter cryohalolentis K5
Geobacillus Y412MC61
Burkholderia phymatum STM815
Desulfovibrio vulgaris Hildenborough
Thermococcus sibiricus MM 739
Roseobacter denitrificans OCh 114
Synechococcus CC9605
Helicobacter pylori P12
Acidimicrobium ferrooxidans DSM 10331

Bacterial species
Aliivibrio salmonicida LFI1238
Sulfolobus islandicus
Chlorobium luteolum DSM 273
Cellulophaga lytica DSM 7489
Haloferax volcanii DS2
Amycolatopsis mediterranei U32
Rhodopseudomonas palustris HaA2
Methanocaldococcus vulcanius M7
Lacinutrix
Parvibaculum lavamentivorans DS 1
Salmonella enterica serovar Typhi CT18
Rhodopseudomonas palustris BisA53
Pseudomonas syringae B728a
Leifsonia xyli CTCB07
Streptomyces violaceusniger Tu 4113
Klebsiella variicola
Chlorobium phaeobacteroides DSM 266
Brucella suis ATCC 23445
Dickeya zeae Ech1591
Laribacter hongkongensis HLHK9
Halorhabdus utahensis DSM 12940
Pseudomonas fluorescens Pf 5
Serratia plymuthica AS9
Salmonella enterica serovar Heidelberg SL476
Chlorobium chlorochromatii CaD3
Burkholderia glumae BGR1
Acinetobacter ADP1
Candidatus Moranella endobia PCIT
Leptospira interrogans serovar Lai 56601
Variovorax paradoxus EPS
Thioalkalivibrio K90mix
Yersinia enterocolitica palearctica
Candidatus Amoebophilus asiaticus 5a2
Rhodobacter sphaeroides ATCC 17025
Coxiella burnetii RSA 493
Haemophilus influenzae PittGG
Methylomicrobium alcaliphilum
Haloarcula hispanica ATCC 33960
Escherichia coli S88
Neisseria gonorrhoeae NCCP11945
Delftia acidovorans SPH 1
Megasphaera elsdenii DSM 20460
Pseudomonas mendocina ymp
Salmonella enterica serovar Paratyphi C RKS4594
Wolbachia endosymbiont Drosophila melanogaster
Halomicrobium mukohataei DSM 12286
Yersinia pestis Pestoides F
Yersinia pestis CO92
Escherichia coli ATCC 8739
Escherichia coli K-12 substr MG1655
Neisseria gonorrhoeae FA 1090

Supplementary Table 4 Parameter settings

minPts (DBSCAN)	epsilon (DBSCAN)	perplexity (BH-tSNE)	theta (BH-tSNE)	dims (BH-tSNE)
8	0.019	40	0.5	3

Supplementary Table 5 Number of contigs, their total length, and run time(s) for the simulated metagenomic data with 100 genomes.

Number of contigs	Total length	Run time
8977	343220185	904.95

4-mer as feature space

we provided a direct comparison of our results with 4-mers as the feature space in our pipeline. The results are shown for 10 and 100 genome datasets.

Supplementary Table 6 Precision, recall, F1 score (%), and the number of clusters for 4-mer as the feature in our pipeline.

Dataset	Precision	Recall	F1 score	Number of clusters
10 genomes	96.14	70.80	81.54	13
100 genomes	95.32	69.56	80.43	98

This shows our feature space (MLBP) performs better.

Real Human Gut Metagenomic Data

Supplementary Table 7 Parameter settings

minPts (DBSCAN)	epsilon (DBSCAN)	perplexity (BH-tSNE)	theta (BH-tSNE)	dims (BH-tSNE)
8	0.02	40	0.5	2

Drosophila Dataset

Supplementary Table 8 Parameter settings

minPts (DBSCAN)	epsilon (DBSCAN)	perplexity (BH-tSNE)	theta (BH-tSNE)	dims (BH-tSNE)
8	0.013	40	0.5	2

Supplementary Table 9 Number of contigs, their total length, and run time(s).

Number of contigs	Total length	Run time
21984	120531818	2541.52

Using the Software

1. 10genome dataset:

$./MrGBP -fa raymeta_10.fasta
loading times: 1.54818
feature selectiom times: 44.9119
svd time: 0.349399
Using no_dims = 2, perplexity = 40.000000, and theta = 0.500000

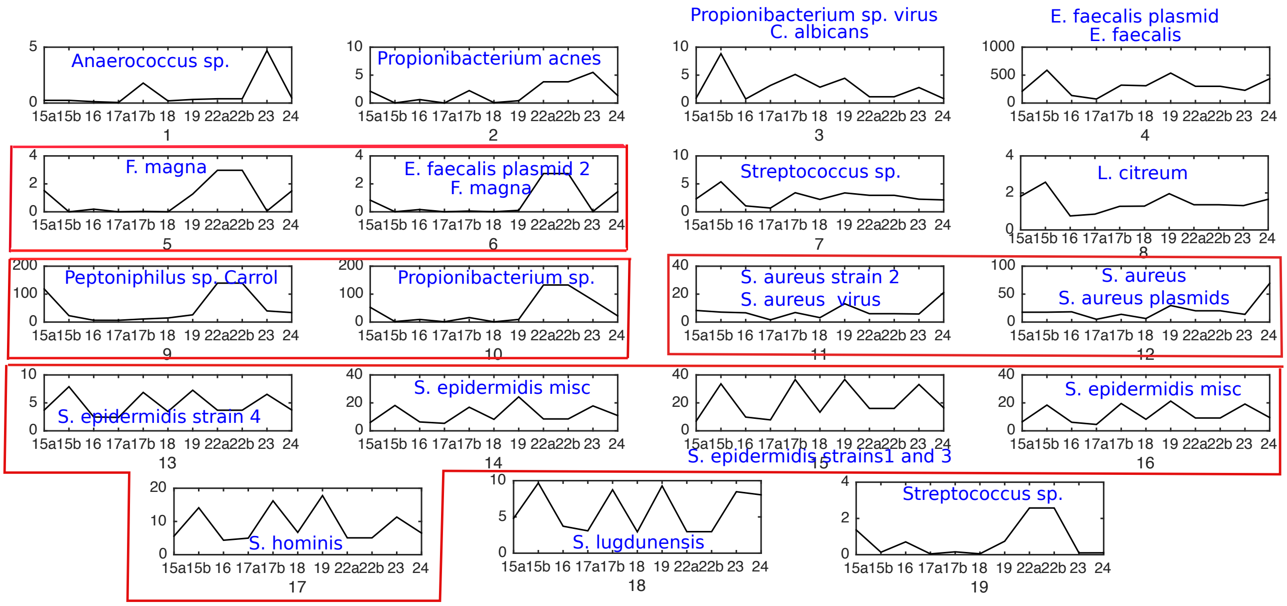

Supplementary Figure 3 Longitudinal abundance patterns of the 19 identified clusters, see Figure 3. The associated species or groups of species are indicated for each cluster. The x-axis corresponds to the longitudinal sampling over nine days[1]. The y-axis corresponds to normalised read coverage. The red box indicate the correlated clusters across longitudinal samples.

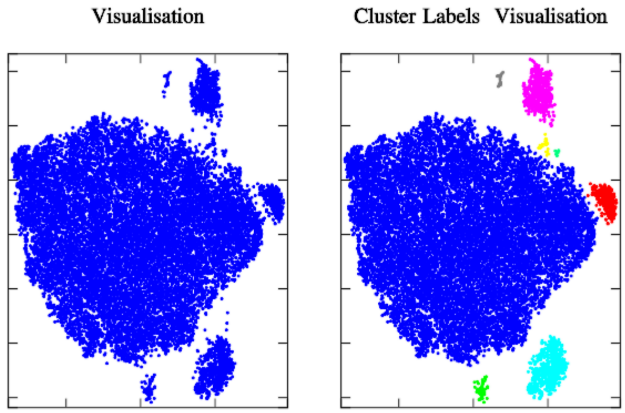

Supplementary Figure 4 Each color shows a cluster defined by our approach on the right hand side figure.

Computing input similarities...
dimension reduction running time: 35.5877
clustering time: 12.056
-out: 12 clusters has been generated.

2. 100 genome dataset:

$./MrGBP -fa Contigs.fasta -no_dims 3
loading times: 60.9531
feature selectiom times: 492.094
svd time: 1.68903
Using no_dims = 3, perplexity = 40.000000, and theta = 0.500000
Computing input similarities...
dimension reduction running time: 334.805

clustering time: 236.07
-out: 78 clusters has been generated.

to have more clusters for the same dataset:

$./MrGBP -fa Contigs.fasta -no_dims 3 -dbep 0.019
loading times: 58.967
feature selectiom times: 488.136
svd time: 1.64656
Using no_dims = 3, perplexity = 40.000000, and theta = 0.500000
Computing input similarities...
dimension reduction running time: 364.718
clustering time: 236.059
out: 116 clusters generated.

3. CAMI low complexity dataset:
$./MrGBP -fa gsa_anonymous.fasta -no_dims 3
loading times: 0.30396
feature selectiom times: 305.768
svd time: 1.4899
Using no_dims = 3, perplexity = 40.000000, and theta = 0.500000
Computing input similarities...
dimension reduction running time: 336.198
clustering time: 156.954
-out: 42 clusters has been generated.

4. CAMI Medium Complexity dataset:
$./MrGBP -fa pooled_gsa_anonymous.fasta -no_dims 3
loading times: 1.09978
feature selectiom times: 966.801
svd time: 4.62844
Using no_dims = 3, perplexity = 40.000000, and theta = 0.500000
Computing input similarities...
dimension reduction running time: 1677.8
clustering time: 1517.35
out: 51 clusters generated (Figure 5):

to have more clusters for the same dataset:

$./MrGBP -fa pooled_gsa_anonymous.fasta -no_dims 3 -dbep 0.016
loading times: 1.13176
feature selectiom times: 972.929
svd time: 4.89773
Using no_dims = 3, perplexity = 40.000000, and theta = 0.500000
Computing input similarities...
dimension reduction running time: 1551.57
clustering time: 1491.36
out: 154 clusters generated (Figure 6).

5. Considering a different contigs minimum length:
$./MrGBP -fa raymeta_10.fasta -mincl 400
loading times: 1.50128
feature selectiom times: 45.2874
svd time: 0.432179
Using no_dims = 2, perplexity = 40.000000, and theta = 0.500000

Computing input similarities...
dimension reduction running time: 45.5465
clustering time: 15.6797

Parameter Setting:

1. Numerical representation of nucleotide sequences: various data representations can affect the results (Supplementary Figure 1 and table 2). It shows that Integer representation has better performance and has been set as the default for the online code.

2. MLBP window length that affects the feature length: Longer feature space my improve the performance similar to other applications. However, increasing the feature length increases the computational complexity/run time (Supplementary Figure 2 and Table 3).

3. Dimension reduction steps using (1) SVD considering various number of eigen factors (Figure 4 and Table 4) and (2) BH-tSNE where the default parameters have been considered except for suggesting keeping 3 dimensions for more complex data can improve the results. Moreover, a review on BH-tSNE parameter settings can be found at: `http://distill.pub/2016/misread-tsne/`

4. DBSCAN parameters: It has two parameters (1) epsilon that indicates the closeness of the points of each cluster to each other and (2) minPts, the minimum neighbours a point should have to be considered into a cluster. Usually these values are not known prior to analysis and there are several ways to select their values. One way is to calculate the distance of each point to its closest nearest neighbour and use the histogram of distances to select epsilon. After selecting epsilon a histogram can be obtained of the average number of neighbours for each point using the epsilon. Some of the samples do not have enough neighbouring points and can be considered as noise. Implementation of the parameter selection is included in spark dbscal (`https://github.com/alitouka/spark_dbscan`). Here, we consider minPts = 8 and epsilon = 0.02 but to have more clusters our suggestion is to reduce epsilon. Visualisation can help to decide if the clusters are satisfactory otherwise the parameters can change.

Moreover, for multi-sample real datasets the coverage information have been added. Consequently, we believe our method does not need many samples to run. Our method performs better for low/medium datasets and also if there is noisy sequences.

References

1. Sharon, I. *et al.* Time series community genomics analysis reveals rapid shifts in bacterial species, strains, and phage during infant gut colonization. *Genome research* **23**, 111–120 (2013).
